# Assessment of Well Integrity for Repurposing O&G Wells for CO_2_ Storage—Essential Safety Considerations

**DOI:** 10.1021/acsomega.5c09944

**Published:** 2026-01-20

**Authors:** Nívia M. Oliveira, Alexandre Szklo, Paulo Couto, Fernanda M. L. Veloso, Isaline Gravaud

**Affiliations:** † Energy Planning Program, COPPE, 28125 Universidade Federal do Rio de Janeiro, Avenida Horácio de Macedo, 2030, Rio de Janeiro 21.941-914, Brazil; ‡ Centre for Energy and Environmental Economics (CENERGIA), Energy Planning Program, COPPE, 28125 Universidade Federal do Rio de Janeiro, Avenida Horácio de Macedo, 2030, Rio de Janeiro 21.941-914, Brazil; § Civil Engineering Program, COPPE, 28125 Universidade Federal do Rio de Janeiro, Avenida Horácio de Macedo, 2030, Rio de Janeiro 21.941-914, Brazil; ∥ C-Questra, Rokin 92-96, 1012 KZ Amsterdam, Netherlands; ⊥ 52810 Bureau de Recherches Géologiques et Minières, 3 Av. Claude Guillemin, 45100 Orléans, France

## Abstract

Wells that develop leaks due to integrity issues can pose serious risks to both the environment and human health, particularly if they release previously captured CO_2_ back into the atmosphere or contaminate freshwater aquifers. Ensuring reliable underground storage, therefore, presents a significant challenge in well integrity. The injected CO_2_ can lead to substantial corrosion of metal materials and cement within the well. Moreover, the quality of cementation may deteriorate due to improper cement placement or changes in its mechanical properties arising from the well’s operational activities. Identifying, quantifying, and mitigating this corrosion, along with assessing cementation quality, are pivotal for maintaining satisfactory well conditions. This study proposes solutions, through a literature review, that focus on material selection and the composition of CO_2_ flow. It emphasizes essential considerations for a preliminary evaluation of both active and suspended wells for their reuse in CO_2_ injection and storage in CCS operations. Conducting a systematic literature review has been crucial in identifying the key factors for assessing wells suitability for CO_2_ injection. The outcome is a flowchart designed for evaluating active and suspended wells, encompassing a qualitative and initial analysis of critical factors such as cement integrity, corrosion, CO_2_ flow composition, and material compatibility. If the recommended actions do not resolve the issues with a well, it should be plugged and abandoned.

## Graphical abstract

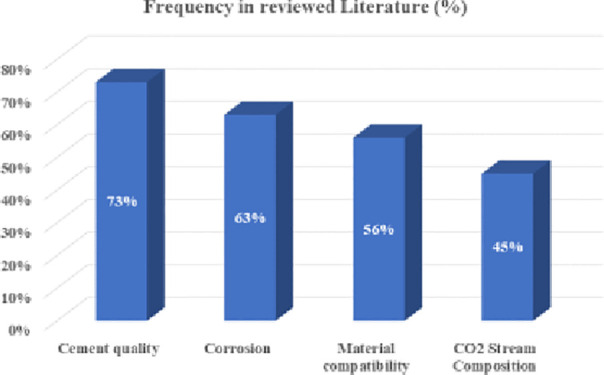

## 1 Introduction

Governments worldwide are committing to net-zero CO_2_ emissions while many oil and gas assets are reaching the end of their operational life and need decommissioning. This creates an opportunity to repurpose these decommissioned wells and fields for CO_2_ injection and storage. Using existing infrastructure can reduce costs and expedite the establishment of carbon capture and storage (CCS) systems.^1^

Saving costs does not apply to every existing wells. Repurposing existing oil and gas wells for alternative energy applications holds promise for sustainability goals, but brings with it a web of economic and technical challenges. The allure of cost savings through predrilled wells is often offset by significant financial outlays required for retrofitting.^2^ CCS technology necessitates its own set of infrastructure modifications, these adjustments are not merely technological integrations but also repairs for wear and tear inflicted by prolonged hydrocarbon extraction.^1,3^

Depleted fields offer opportunities to reuse production sites, wells, pipelines, and platforms, along with valuable reservoir data. However, a site-specific evaluation is necessary, ideally early in the decommissioning planning to prevent additional costs and integrity issues. The risk of containment loss is high in both legacy and new wells, especially where breaches through the caprock may occur. Some wells, previously plugged and abandoned under older regulatory standards, may need reassessment if there’s a chance they will contact CO_2_ during or after injection. Re-entering these wells is feasible onshore but can be very costly offshore^1,4^ .

According to Bai et al.,^5^ evaluating accessible wells is relatively straightforward. In contrast, evaluating abandoned wells poses significant challenges due to limited available data for direct assessments, rendering conventional methods inapplicable. Moreover, reopening and investigating an abandoned well incurs substantial costs.

To ensure the long-term viability of CO_2_ storage, it is crucial to assess the reservoir’s integrity and existing wells. This includes identifying and fixing any high-risk pathways for CO_2_ migration and evaluating well construction for compatibility with low pH fluids, since carbonic acid can corrode carbon steel when free water is present. Recognizing potential corrosive elements like free water, NO_*x*_, SO_*x*_, H_2_S, and O_2_ is important for material specifications. Additionally, accurately accounting for net volumes of CO_2_ stored, apart from those purchased and recycled, is vital for acknowledging sequestered CO_2_.^3,6,7^

The monitoring requirements for geological storage focus on ensuring that injected CO_2_ remains contained within the target formation, without lateral or vertical migration that could affect other resources or the surface. In a CO_2_-EOR operation, numerous wells and historical oil field activities complicate baseline establishment for monitoring. The presence of abandoned, suspended, or active wells increases leakage risks, necessitating risk assessment and mitigation, which may involve additional monitoring or reabandonment at significant costs.^3,8^

Reutilizing existing wells is a powerful solution to prevent further breaches through the caprock while breathing new life into well infrastructure. Instead of decommissioning these wells and constructing new ones, which risks compromising the integrity of the sealing rock, it should prioritize this approach as it conserves resources and ensures the stability of the reservoir. By embracing this strategy, it paves the way for a sustainable future, maximizing the current assets to guarantee both efficiency and safety. While not every well may be suitable for reuse, it is essential to conduct a thorough assessment before moving forward with new drilling. This must allow a comprehensive economic evaluation to avoid the trap of perceived savings morphing into escalating costs.

Recent studies have explored critical issues surrounding well integrity for CO_2_ geological storage (e.g., refs 3,5,9 ). However, a direct methodology for assessing both active and suspended wells, particularly regarding challenges like corrosion, cement quality, CO_2_ composition, and material requirements, has yet to be established. In response to this gap, the current study embarks on an important mission to evaluate the feasibility of repurposing oil and gas wells for effective CO_2_ storage.

Through an in-depth literature review and insights gleaned from various research initiatives and field operations, we meticulously compile existing procedures for well reuse in CO_2_ storage. We also highlight the essential components that underpin safe and efficient operations. Furthermore, this study proposes a standardized workflow for the assessment process, drawing on relevant standards successfully implemented in CO_2_-EOR by the petroleum industry. The guidelines outlined in ISO27914 will serve as a foundational benchmark for advancing geological CO_2_ storage methodologies and will inform our final recommendations with clarity and precision.

## 2 Materials and Methods

For this systematic review, searches were conducted across several databases, including ScienceDirect, OnePetro, ACS Publications, Scopus, Google Scholar, book chapters, and Technical Reports. The search utilized various combinations of keywords such as ‘CO_2_ injection wells’, ‘CO_2_ well integrity’, ‘leakage risk factors’, ‘CO_2_-EOR’, and ‘CCS commercial scale projects’. The review focused on papers published from 1959 to 2025, encompassing primarily peer-reviewed articles (40 papers), along with some conference papers (11 papers) and technical journal and reports (22 papers and reports). Studies that addressed other forms of carbon storage were excluded. Ultimately, a total of 73 references met the eligibility criteria after a thorough screening of titles, abstracts, and full texts.

A spreadsheet was developed to catalog bibliographic data, including author, year, paper originality, and key findings, while evaluating CO_2_ leakage risks. The criteria noted in the papers and technical reports were organized into thematic categories: corrosion, cement, material requirements, and CO_2_ stream composition. Issues related to cement, such as its composition, inadequate cementation, and the necessity for high-quality cementation, were mentioned in 77% of the studies. Corrosion issues were referenced in 67% of the papers, material requirements in 59%, and CO_2_ stream composition in 48% (Figure 1).

**Figure 1:**
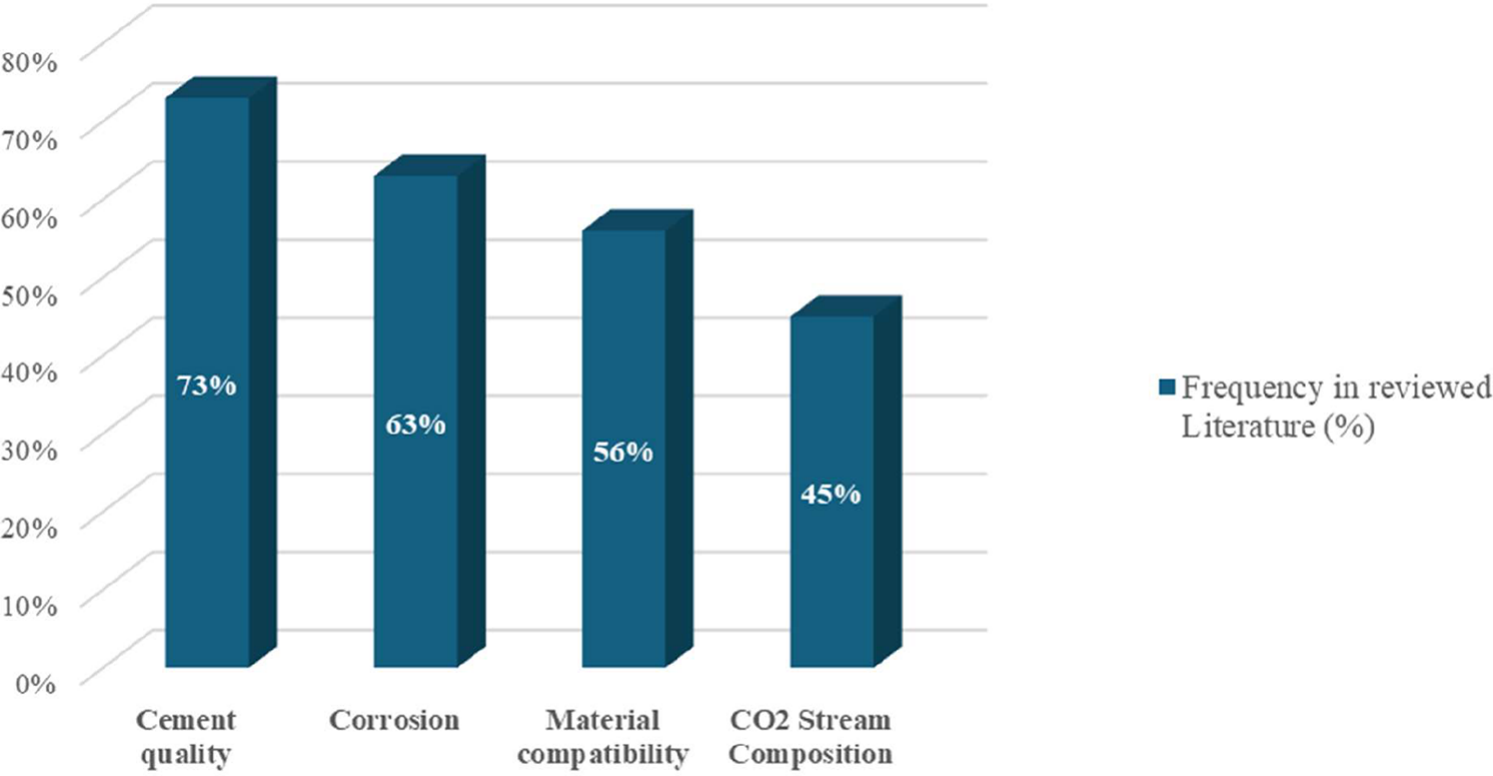
Percentage of studies addressing each factor (own analysis of the systematic review data).

## 3 Previous Relevant Assessments for CO_2_-EOR and Well Integrity

Well integrity, as defined by Crow et al.,^10^ refers to the isolation of geological formations, preventing fluid movement. During well construction, a steel casing is inserted into the wellbore and secured by a Portland cement sheath. Injecting CO_2_ can further compromise well integrity due to potential corrosion of the steel and carbonation of the cement, which affects the casing-cement-host rock region and any cement plugs.

In the context of CO_2_-EOR operations, the wells can either be newly drilled or converted from existing production or water injection wells into CO_2_ injectors. Numerous studies have explored the conversion of existing wells for this purpose. Notably, Folger and Guillot,^11^ Power et al.,^12^ and Bowser et al.^13^ have documented significant field redevelopment initiatives related to this process.

Bowser et al.^13^ examined techniques to convert water injection wells into CO_2_ injectors for enhanced oil recovery (EOR) in the Maljamar Cooperative Agreement in southeast New Mexico. They noted that inadequate casing and cementing practices could hinder long-term recovery projects. To avoid redrilling and reduce costs, they proposed methods to minimize water loss, enhance fluid distribution, and use CO_2_-resistant injection equipment.

For that, their study indicates the use of resin-coated sand in the recompletion process, where the resin would act to decrease the enlarged hole interval and help return to the original diameter. This hole enlargement can be created for several reasons, for instance, by shooting of pay zone for well stimulation, by erosion due to high velocities, or by geologic conditions. Thinking about corrosion, they selected fiberglass liners because they are relatively drillable and corrosion-resistant. The plan for this study was to utilize a continuous CO_2_ injection scheme for believing that corrosion should be less severe than with a WAG (Water-alternating-gas). The BOP wellhead was also an operational concern due to the cost of CO_2_. Historically, before repairing a packer or casing, it was necessary to backflow large volumes of water, but this operation is extremely expensive in the case of CO_2_. This way, they designed a wellhead that allows pressure on the tubing/casing annulus to be contained while nipple-upping a snubbing stack. Thus, the CO_2_ remains in the well, saving time and money. One of the conclusions stated that using these innovative techniques to recomplete the old injection wells at the MCA unit has resulted in 50% cost savings over redrilling the wells.

In the North Ward Estes (NEW) field, Texas, Power et al.^12^ evaluated various methods to convert 165 water injection wells to CO_2_ injectors. Discovered in 1929, the field began waterflooding in late 1954, and several tertiary pilot projects, including in situ combustion and polymer injection, took place since 1975. The viability of a CO_2_ flood was explored in 1985, yielding positive results and initiating well conversions. This involved replacing, cleaning, or treating existing structures, repairing casing leaks, sidetracking junk, and mudding sloughing shot holes.

The team responsible for this project looked after many wells drilled years ago, which did not have cement on the production casing strings. Thus, long intervals were exposed to highly corrosive water flows from the formation. Some experiments were done to adjust the cement to give enhanced CO_2_ resistance. According to them, remedial-cementing success can only be improved by meticulously observing cement and additive quality assurance, accurately calibrating testing equipment and procedures, and deliberate placement techniques. After years of injection, the liners exposed to corrosive water presented severe corrosion problems at various depths. Fiberglass or steel liners were found in these points to prevent or postpone formation-fill problems, and thus, they cleaned up these wells, removing liners to assess the real well conditions. In certain cases, the liners had deteriorated to the point where a mill orbit could be utilized to drill out the remaining metal.

Folger and Guillot^11^ outlined methods for designing a miscible CO_2_ flood at the Sundown Slaughter Unit (SSU) in Texas, including reservoir characterization and field preparation. Discovered in April 1937, the field initially produced under gas drive, with waterflood operations starting in the late 1950s. By 1980, it was in the mature stages of waterflooding. Nearby units had already implemented CO_2_ flooding, allowing SSU to leverage existing CO_2_ supply pipelines and processing infrastructure. CO_2_ injection at SSU began on January 4, 1994.

Many injectors drilled in the 1930s and 1940s needed to be upgraded to last another 30 years under rigorous CO_2_ injection service. They underwent important modifications to the CO_2_ flood. Depending on the extent of corrosion, 10 to 15 feet of surface casing was removed and replaced with new casing due to external corrosion found near ground level. In some cases, the production casing showed signs of corrosion and was cut and replaced. The CO_2_ properties and frequent makeup and breakout have caused collar leaks higher than expected. Fiberglass-lined tubing was included in the installations, which should result in a reduction in failure rates. Packers were fitted with CO_2_-compatible elastomers in packing elements and were internally nylon-lined and externally nickel-coated.

Modification was minimal for the wells that were produced. Each was equipped with a rod BOP and hard-faced polished rod. Once they failed ones with a nickel-carbide barrel and valves replaced downhole rod pumps. Several suitable materials were developed for reusing existing wells to CO_2_ flooding. Still, a huge advantage of this Unit was the great data available on other CO_2_ projects in the same field, Slaughter. This way, they immensely influenced the project design.

The evolution of CO_2_ injection has a rich history, beginning with the first field test in 1964 at the Mead Strawn field in Texas, developed by Holm and O’Brien.^14^ They aimed to examine the effects of CO_2_ injection, followed by carbonated water and brine, involving four injection wells and two production wells. Although they encountered corrosion issues early on, their primary focus was on field productivity. Despite treating the brine for bacteria and corrosion, issues persisted, leading to the addition of a corrosion inhibitor and bactericide, which reduced the corrosion rate. However, mechanical failures in plastic coatings continued to be a problem. Severe corrosion in casings caused shutdowns, but production rates remained steady, while oil flow rates decreased due to waterflood impacts. Overall, the project demonstrated a significant oil production increase of 53 to 82% in optimal areas compared to water-only flooding, aligning with Holm’s^15^ earlier laboratory findings. Subsequent laboratory tests and pilot projects followed to further this research.

The world’s first commercial CO_2_-EOR injection project was initiated in the early 1970s on the SACROC (Scurry Area Canyon Reef Operators Committee) oilfield in the Gulf of Mexico.^16–20^ This recovery project included converting over 200 producing wells to CO_2_ injectors, expanding surface facilities, and constructing and operating the CO_2_ pipeline-compression system. Therefore, studies were necessary on what type of material to use under operating conditions to prevent corrosion, material wear, and leakage. Cement linings for steel pipe were also tested to determine if they would deteriorate on exposure to high-pressure CO_2_ or be subject to loss of the cement’s water of crystallization to the ultradry CO_2_. At supercritical state pressures, the cement linings resisted dry CO_2_ attack satisfactorily. It was found minimal corrosion in the dry CO_2_ portion of the injection system and maximum in meter runs, wellheads, and tubing, which are subject to alternating water-CO_2_ slugs.^16,17,21,22^

Newton and MacClay^22^ presented a great evolution in materials technology for CO_2_ injection systems for the SACROC field. Faced with the corrosion problem at CO_2_-water injection wells, some investigations were done. Initially, meter runs were constructed of plastic-coated carbon steel, and valves had plastic-coated carbon steel bodies with 316 SS trim. They were subjected to severe corrosion at any point of coating damage, where 316 SS was used, no corrosion was observed. After this, meter runs were constructed entirely of 316 SS pipe and valve. The injection wellheads were equipped with 410 SS wellheads and 410 SS valves. Nevertheless, these wellheads were selected and ordered before the decision to WAG operation, as they were subjected to severe pitting-type corrosion due to suspended matter in the produced injection water. A replacement program using all 316 SS wellheads was planned to prolong the useful life of many of the wellheads.

Another point Newton and MacClay^22^ presented, relates to the injection wells, which were equipped with a thin film epoxy-modified phenolic type of plastic-coated tubing set on plastic-coated double-set packers. Up to 25% of the injection wells had tubing pulled and inspected each year due to tubing leaks or for workover purposes. Tubing reclaimed as plastic-coated injection tubing averages 53%, with 47% being downgraded for another service because of many failures. However, the primary cause of failure has been mechanical damage occurring during hauling, running, and pulling. Given this, there was a need to modify some handling and installation procedures to avoid mechanical failure. Table 1 summarizes the materials commonly utilized for CO_2_ injection well components in the USA projects.

**Table 1:** Common Materials and Considerations for CO_2_ Injection Well Components, Synthesized from Field Studies and Industry Reports (Synthesized and Adapted from Refs 11,22–24 )

component	common material of construction (MOC) and specifications	key considerations and alternative materials
surface piping/meter runs	316 stainless steel (SS), fiberglass	316 SS provides full corrosion resistance in WAG operations. Carbon steel with a coating is susceptible to severe pitting at any point of coating damage.
wellhead/Christmas tree	316 SS trim (carbon steel or 316 SS), nickel, Monel	410 SS is susceptible to pitting corrosion in WAG service. 316 SS is the preferred upgrade. Elastomer seals must be CO_2_-compatible.
tubing	GRE-lined carbon steel, internally plastic-coated (IPC) carbon steel, corrosion-resistant alloys (CRA)	plastic coatings (epoxy-modified phenolic) are effective but prone to mechanical damage during handling. 13Cr steel is resistant to CO_2_ corrosion in wet conditions if free water is limited.
tubing joint seals	seal rings (GRE), coated threads and collars (IPC)	critical to prevent leakage at connections. Design must account for thermal and pressure cycling.
packers	internally coated hardened rubber (e.g., Buna-N, 80–90 durometer), Nickel-plated wetted parts	elastomers in packing elements must be CO_2_-compatible. Metal parts exposed to the fluid stream require corrosion-resistant coatings or materials.
cement	API class G or H cement with pozzolanic additives (e.g., silica fume), calcium aluminate cement (CAC), acid-resistant specialty cements	Portland cement carbonates in contact with CO_2_-saturated water but can retain integrity if well-placed. Additives (elastomers, fibers) improve flexibility and reduce permeability. CAC shows superior mechanical and microstructural performance under high-temperature CO_2_ conditions.

EOR operators can achieve wellbore integrity similar to conventional oil and gas wells through suitable technologies. Evidence suggests a low risk to the geologic integrity of receiving formations, supported by 30 years of samples from a CO_2_ injection well, which confirm the preservation of near-reservoir and cement-sealing integrity.^25^

Gill^26^ noted that after ten years, the SACROC field operations validated the original component designs, with some necessary operational improvements. The system’s complexity and the interrelationship of components were emphasized.

Carey et al.^25^ later studied the Portland-cement-based wellbore system of a 55-year-old well exposed to CO_2_ at 54 °C and 18 MPa for 30 years. Samples collected from above the reservoir-capprock interface showed up to 1 cm-thick carbonate deposits along the cement-casing and cement-shale interfaces. Notably, the Portland cement maintained its structural integrity despite CO_2_ exposure, indicated by carbonate precipitate and heavily carbonated cement adjacent to the shale caprock.

Barlet-Gouédard et al.^27^ and Duguid and Scherer^28^ compared their studies to this study developed by Carey et al.^25^ Barlet-Gouédard et al.^27^ noted that in conventional cement, there is a formation of microannuli between cement and the casing, as well as between cement and rock formation, which compromises its integrity, while in cement with an additive, no microannuli were detected. These observations are inconsistent with Carey et al.,^25^ which showed that Portland cement retained its structural integrity after 30 years near the presence of CO_2_. On the other hand, Duguid and Scherer^28^ obtained similar results to those found in the SACROC field.

Crow et al.^10^ investigated a 30-year-old well in the Dakota sandstone formation, which has been consistently exposed to CO_2_. Unlike other studies, this well has a known quantity and saturation of CO_2_, and its reservoir may not provide adequate buffering against CO_2_’s corrosive effects. The barrier system has performed well over three decades, with cement still effective as a hydraulic barrier despite carbonation effects diminishing with distance from the reservoir. The carbonated cement exhibited higher permeability and porosity but lower mechanical strength compared to lab-cured cement. The casing was in good condition, with minimal fluid flow along the casing-to-cement interface. While both studies indicated that cement changes due to CO_2_ exposure could still ensure effective CO_2_ migration barriers, laboratory research noted that well cement used in enhanced oil recovery (EOR) applications showed a loss of compressive strength and structural integrity, with greater reactivity in supercritical CO_2_ under dynamic conditions.^29,30^

Four chemical environments can be observed when injecting supercritical CO_2_ into deep saline aquifers and hydrocarbon reservoirs:

- Dry supercritical CO_2_ can be found near the injection well;
- a CO_2_ plume contains wet supercritical CO_2_;
- CO_2_-rich water can be found whose composition is similar to that of the CO_2_ plume;
- the original brine contains an insignificant amount of dissolved CO_2_.

The second and last Environments are the most important scenarios for studying cement carbonation in wells that are not injectors of CO_2_, according to Zhang and Bachu.^31^

In reservoir conditions, chemical reactions lead to cement carbonation when supercritical CO_2_ and/or CO_2_-saturated formation water is in contact with cement.^32^ CO_2_ in the form of carbonic acid can chemically react with many commonly used wellbore materials such as casing or cement.^33^ But the carbonation rate in cement depends on the chemical and physical properties of cement, such as portlandite content, calcium silicate hydrate content, porosity, and tortuosity, as well as the processing conditions such as curing time, temperature, pressure, and fluid flow rate.^34,35^

Kutchko et al.^32^ analyzed cement degradation, focusing on curing under different pressure and temperature conditions. All experiments were reported under static conditions. They observed that the cement samples were exposed to identical CO_2_ sequestration conditions (50 °C and 30.3 MPa). Upon aqueous CO_2_ exposure, they formed a well-defined band of calcite and had the smallest amount of penetration from the acid attack. They also state that changes in cement morphology at such high temperatures could have made it much more vulnerable to acid attack by CO_2_.

Several experimental, numerical, and field studies have been carried out to observe the degradation rate in different ways under different experimental conditions, considering changes in cement’s physical and chemical attributes. In addition, large-scale projects and assessments focusing on wellbore integrity related to reusing existing oil and gas wells from depleted fields have been developed. Some projects and assessments will be highlighted in the following items. All these efforts present complementary, similar, and even contrasting results. Diversification in the conclusions fosters the need to develop a relevant methodology for assessing the risk of CO_2_ leakage in existing wells and give some recommendations related to well integrity data management. This requires comprehensive monitoring and proactive warning methodology, identifying integrity issues and alerting operators before major failures arise.

Table 2 summarizes the most relevant studies, highlighting their main critical observations, proposed suggestions, and challenges categorized into corrosion, CO_2_ composition, cement quality, and material requirements.

**Table 2:** Summary Table of Relevant Studies in the Evaluation of Wells for Reuse in CO_2_ Injection

**study**	**critical/observed scenario**	**proposed solution**	**challenge category**
**14**	• corrosion problems in the system	• corrosion inhibitor and bactericide were promptly introduced into the system	corrosion
**22**	• corrosion problem at CO_2_-water injection wells	• meter runs were constructed entirely of 316 SS pipe, valve, and wellhead	corrosion and CO_2_ composition
**13**	• casing and cementing practices were often inadequate for a long waterflood life would be unacceptable for a long-term tertiary recovery project	• use of resin-coated sand in the recompletion process	material requirements, cement quality, and corrosion
		• use fiberglass liners because they are relatively drillable and corrosion-resistant	
**12**	• long intervals of the production casing strings did not have cement, thus, were exposed to highly corrosive water flows from the formation	• experiments were done to adjust the cement to give enhanced CO_2_ resistance	material requirements, cement quality, and corrosion
	• liners exposed to corrosive water presented severe corrosion problems at various depths	• Fiberglass or steel liners were found in these points to prevent or postpone formation-fill problems, and thus, they cleaned up these wells, removing liners to assess the real well conditions	
**11**	• CO_2_ properties and frequent makeup and breakout have caused collar leaks higher than expected	• fiberglass-lined tubing was included in the installations, which should result in a reduction in failure rates. Packers were fitted with CO_2_-compatible elastomers in packing elements and were internally nylon-lined and externally nickel-coated	CO_2_ composition, material requirements, and corrosion

## 4 Relevant Awareness of Reusing Oil and Gas Facilities for CO_2_ Storage

When considering CO_2_ injection for storage, it is essential to evaluate factors like operational lifetime. Active wells possess the infrastructure to address integrity issues, while abandoned wells pose greater risks for maintaining integrity, especially in CO_2_ storage. Additionally, chemical conditions during CO_2_ injection differ from those in traditional production wells, which are designed for depleted pressures rather than overpressured environments. Although experience from CO_2_ injection in tertiary oil recovery is helpful, storing CO_2_ presents unique challenges in fluid management, pressure, and long-term integrity requirements. For CO_2_ storage to be accepted as a safe greenhouse gas control method, it must be demonstrated that the well is leak-free.^36^

For the CCS to be successful, the storage environment must meet three basic conditions: capacity, injectivity, and containment. It is crucial to remember that the last condition is critical because CO_2_ leakage puts other resources at risk of contamination, such as potable groundwater, vegetation, animal life, and human health.^37^ There are natural pathways for CO_2_ leakage, such as open fractures, active or reactivated faults, and pathways made by humans, mainly wells. Oil and gas wells may provide leakage pathways due to either mechanical defects developed during well drilling, completion, and/or abandonment or to chemical degradation of well cement and/or casing.^31,38^ As shown in Figure 2, CO_2_ can leak along the well in several ways. The leakage can happen at different interfaces between the materials, such as the steel casing cement interface (Figure 2a), the cement plug steel casing interface (Figure 2b), and the formation cement interface (Figure 2f). Additionally, CO_2_ can leak through fractures in the cement (Figure 2c,e) and fractures in the steel casing (Figure 2d).^39^ Apart from these smaller-scale features, leakage can also occur when the wells are only cemented over a short interval or when the cement sheet does not uniformly cover the entire circumference of the well. Furthermore, casing corrosion can lead to casing failure and large leakage pathways.^40^

**Figure 2:**
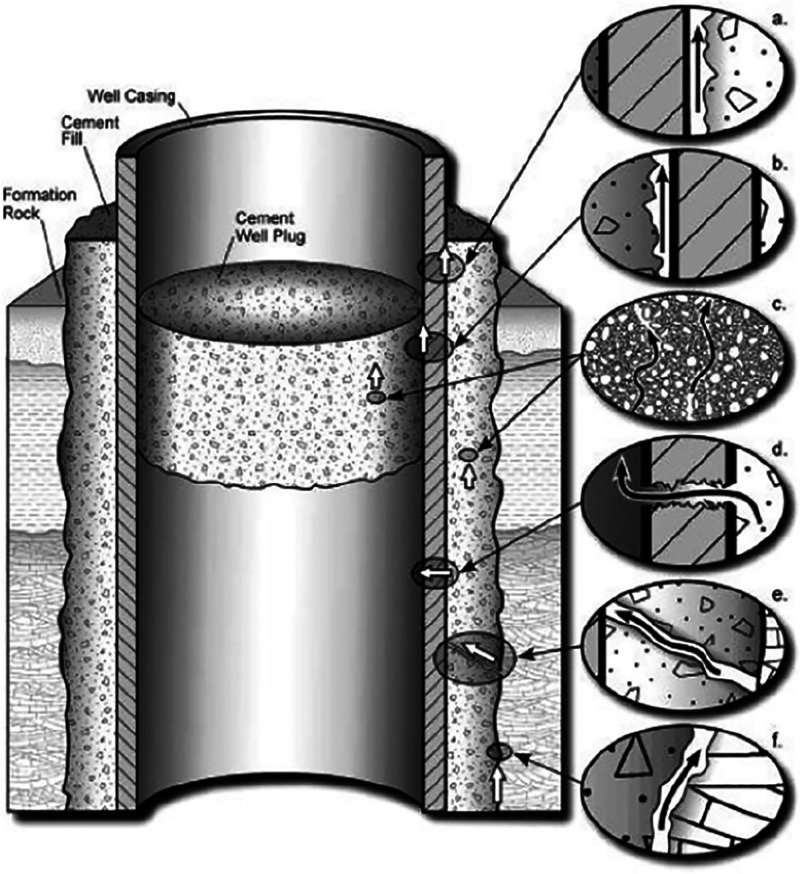
Illustration of possible CO_2_ leakage paths. (a) steel casing cement interface; (b) cement plug steel casing interface; (c) fracture in the cement; (d) fractures in the steel casing; (e) fracture in the cement; (f) formation cement interface (Celia et al.).^39^ Licensed by Elsevier and Copyright Clearance Center.

Well integrity in oil fields and CO_2_ storage has been explored in several studies (e.g., refs 39,41,42 ). Although CO_2_ storage research is relatively recent, the corrosion mechanisms and leakage pathways remain similar to those in oil fields. Depleted oil and gas reservoirs present challenges for CO_2_ storage due to the numerous existing wells, which can impact storage security.^36^

Leakage pathways may form in cement plugs or annular regions.^10,43^ Understanding CO_2_’s effects on Portland cement under reservoir conditions is crucial, as even properly placed cement can fail due to changes in downhole conditions. These changes, influenced by casing pressure and temperature, can induce stresses that compromise the cement sheath, potentially causing displacement, tension or compression failures, and creating micro annuli and radial cracks.^44,45^

The cement annulus is among the factors affecting the potential for well leakage, where fluid migration could occur through fractures, channels, or pore space. This factor was analyzed in a study,^46^ which examined the reaction of CO_2_ with cement along pre-existing leakage paths. In comparison, fractures present in cement can either close (self-seal) or open upon carbonation. The usual cause of self-sealing is the precipitation of CC̅.^47^ It could also result from cement swelling or deformation.^48^ Fracture opening is usually caused by the dissolution of cement compounds.^49^ Chavez Panduro et al.^46^ observed that the flow conditions and history severely impact the CO_2_ leakage risk from a wellbore. A high CO_2_ flow rate could severely degrade the cement. Conversely, small CO_2_ flow rates promote the formation of the carbonated zone and precipitation of CaCO_3_ in large cavities.

There are limitations in the literature on evaluating the reuse of wells on a field scale for CO_2_ storage. Some of them will be cited in the following. Hawkes et al.^50^ presented one relevant study on a field scale that overviewed the wellbore integrity, reviewing the literature to better understand the impact of wellbore integrity on the long-term security of CO_2_ storage reservoirs. The applications of their efforts would be at Weyburn and Midale fields, which are located adjacent to one another in southeast Saskatchewan, Canada. The oil production from these fields began in the 1950s, with full-scale CO_2_ flooding in 2000 (Weyburn) and 2005 (Midale). Given that there are over four thousand wellbore penetrations in these fields combined, effective implementation of CO_2_ storage requires understanding the hydraulic properties of these wellbores, their responses to CO_2_ exposure, appropriate tools for monitoring their performance, and knowledge of appropriate remediation options.

Among the topics addressed by Hawkes et al.^50^ were issues related to wellbore annular seal, cement degradation in CO_2_, steel corrosion in CO_2_, and operational issues associated with CO_2_. Cement integrity was identified as the most important indicator of wellbore integrity as a major conclusion from their study. However, they also recognized the importance of the roles played by casing, wellbore completion practices, and the history of well-operational activities. None of these practices can satisfy good well integrity acting alone, and acting on a set of essential practices is necessary.

Raza et al.^51^ outlined guidelines for selecting wells suitable for injection and storage in depleted oil and gas reservoirs in Malaysia’s largest gas field. The methodology consists of several steps. First, well locations are prioritized based on integrity analysis, using seismic interpretations to avoid faults and fractures that could lead to CO_2_ leakage due to increased injection pressure.^52^ Key criteria include well status, depth (greater than 800 m), and casing and cement integrity, assessed through production logs. The second step involves analyzing lithofacies and petrophysical data near the wellbore for heterogeneity, porosity (over 20%), and permeability (over 100 mD). Suitable storage requires a reservoir thickness of more than 50 m.^51^ Finally, the presence of fractures near the wellbore must be assessed to ensure that injection pressure remains below the original reservoir pressure, preventing caprock fracturing and potential leakages.

Nygaard^40^ conducted a study to develop a workflow for ensuring long-term well integrity for new CO_2_ injection and monitoring wells, while also assessing the leakage risk of existing wells in the Wabamun field, Canada. The study aimed to determine if wells required reabandonment or workover due to unacceptable conditions. It established requirements for wells drilled after 2003, emphasizing the protection of nonsaline water sources and hydraulic isolation between porous zones. The study verified whether wells were properly cased and abandoned or plugged, checked the type of cement used in open-hole plugs, and assessed if perforations and hydraulic stimulation created fractures that could increase the permeability of the cement sheath.

A number of the technical specifications and evaluation criteria applied to repurpose wells for CO_2_ injection will also be relevant for dedicated monitoring wells. Depending on the position of potential wells in relation to the expected chemical, pressure, and temperature conditions surrounding the reservoir, the significance of certain material compatibility requirements may be diminished. Factors to consider include:

- The specific aim and extent of well-based monitoring;
- Whether the well will interact with the CO_2_ plume or be influenced by acidic fluids;
- The anticipated degree of pressure increase at the location of the well;
- Closeness to the area impacted by thermal stresses near the injection wells.

It is important to remember that even if the well is not utilized as a dedicated monitoring well, chances for collecting monitoring data may arise during workover activities and during later routine maintenance.^53^

## 5 Projects for Large-Scale Involving CO_2_ Storage and Well Integrity in Depleted Oil and Gas Fields

Additionally, numerous projects focus on CO_2_ storage in depleted oil and gas fields, where it is essential to assess well integrity. Over the last year, there has been notable advancement in policies, laws, and regulations specific to CCS in numerous countries, which is evident in the rise of commercial CCS projects that are currently operational, under construction, or in the developmental stage. In various regions around the globe, it is becoming more apparent that there is a connection between the prompt establishment of national policies and regulatory frameworks and the growing number of CCS projects in the pipeline.^54^

The CCS project pipeline has experienced robust growth in 2025, highlighting another year of consistent advancement. Following last year’s report, an unprecedented number of projects have moved into the operational and construction stages. Currently, 77 projects are in operation, with 47 more in the construction phase. Since 2017, the planned global capacity has been increasing at a compound annual growth rate exceeding 30%.^54^

This present study highlighted three projects, such as Kingsnorth CCS, Peterhead CCS, and REX-CO_2_. These initiatives showcase a variety of outcomes related to success, failure, innovation, and collaboration while also addressing challenges such as financing, public acceptance, and scalability. Collectively, they have contributed to the ongoing development of key references in the field. Unfortunately, the first two projects were not implemented due to insufficient government funding. However, several technical reports were produced during the Front-End Engineering and Design phases. The REX-CO_2_ project built upon insights gained from these earlier initiatives to develop a tool specifically designed for assessing the well integrity of CO_2_ injection and storage sites.

The Kingsnorth CCS project, led by E.ON UK, involved various partnerships and government support but was canceled in 2013 due to changes in UK energy policy that withdrew funding for carbon capture and storage (CCS) in coal plants. Consequently, E.ON decided to close the Kingsnorth power plant in 2013. Key technology partners included National Grid for CO_2_ transportation and Shell for precombustion capture technology. The study aimed to enhance knowledge related to CCS design and execution at Kingsnorth, providing an initial estimate of commercial feasibility. The project planned to install two 800 MW generating units and a 300 MW postcombustion carbon capture plant, along with CO_2_ transportation infrastructure to the Hewett gas field. However, it faced funding issues and ultimately closed in July 2015.^55,56^

An assessment of the Hewett field wells included 28 production and 5 exploration wells, but the exploration wells were inaccessible, raising uncertainty about potential leak paths. The assessment concluded that while production wells could be converted for CO_2_ injection, they were unsuitable due to outdated infrastructure and integrity concerns. Any abandoned wells would need to be addressed with CO_2_-resistant methods, though feasibility and costs remain uncertain.^55^

The Peterhead CCS project, funded by the UK CCS Commercial Scale Demonstration Programme and carried out by Shell U.K., aimed to demonstrate the commercial-scale feasibility of capturing about one million tonnes of CO_2_ annually for 10 to 15 years from the flue gas of a gas turbine at Peterhead Power Station. The captured CO_2_ would be compressed and conditioned for transport via a new pipeline to the Goldeneye platform, where it would be injected into a depleted hydrocarbon reservoir over 2 km beneath the North Sea seabed. This initiative also aimed to explore North Sea storage potential and provide insights into capture technologies and CO_2_ transportation.^57^

Funding for the FEED (Front End Engineering Design) studies of the Peterhead CCS project was granted in February 2014 and completed by December 2015. However, the UK government’s withdrawal of funding for the CCS Commercialization Competition led to the project’s cancellation that same year. Despite this, the project was technically feasible, as shown by competitive construction bids.^58^

To transition the platform and pipeline for CO_2_ injection, several modifications were necessary. Evaluating well integrity under CO_2_ exposure was critical for safe storage.^59^ An assessment of the wells near the Goldeneye field involved two phases: evaluating 13 existing exploration wells and five proposed injection wells. Only four of the five suspended wells needed work, with three designated for injection and one for monitoring; the fifth was planned for abandonment.^60^

An assessment of abandoned exploration and appraisal (E&A) wells revealed that eight were outside the expected CO_2_ migration area and had no connection to the reservoir. One well was near the maximum predicted CO_2_ migration distance but also had no contact with the reservoir. The barriers were found to be in good condition, with a very low risk of leakage. Two E&A wells had good seals and were in contact with the reservoir, while two others presented a low leakage risk, with CO_2_ migration expected to take over 20 years, allowing for monitoring and remediation.^61^

In evaluating the conversion of Goldeneye wells from hydrocarbon production to CO_2_ injection, attention was given to materials, casing design, cement, and pressure management. The casing strings on the Goldeneye Platform are made of carbon steel, mostly protected by 13 Chrome (13Cr) tubing, which is resistant to CO_2_ corrosion in wet conditions (Shell). The hydrocarbon gas in Goldeneye contains 0.4% CO_2_, with negligible corrosion observed in the 13% Cr components during production.^60^

The design of the casing has been thoroughly checked, considering the anticipated CO_2_ pressures, temperatures, and volumes. After careful examination, no problems have been found with the casing design. Additionally, any potential compatibility issues between carbon steel and CO_2_ can be addressed as long as the exposure of the casing to wet events is limited to no more than 165 days over a 15-year period.^60^ In carbon steel tubulars, CO_2_ corrosion can be prevented by managing the water content of the CO_2_ to avoid free water formation and prevent wet excursions. To ensure this, the water content in CO_2_ should be kept below 20 ppmW.^24^

The impact of injecting CO_2_ into the cement used in the Goldeneye wells, including E&A wells, has been thoroughly reviewed. As part of workover operations, the project planned to run CBL to better understand the cement’s current integrity. The results of the review showed that the cement composition and volumes placed are consistent with good practices. During dry CO_2_ injection, carbonic acid is not formed, eliminating the potential for chemical reaction with Portland cement. This is the main cause of cement degradation, which is no longer a concern during dry injection. However, there are cases where water may be present around the wellbores later in the wells’ life, which may lead to carbon acid degradation. Nevertheless, from field results, research, software modeling, and experimental data, the existing Portland cement is suitable for CO_2_ injection and storage. It is recommended that the cement quality and placement be evaluated by means such as CBL (Cement Bond Logging) and USIT (Ultrasonic Imager Tool).^60^

To minimize cement degradation, it is crucial to pump dry CO_2_, avoid water during injection to prevent carbonic acid formation, and ensure proper cement placement with good spacers and centralization to prevent voids. Using balanced cement plugs can help avoid stringers and channeling, while keeping excess water minimal and utilizing an inert filter can close interstitial spaces. Follow best cementing practices such as slurry testing, using fresh cement, controlling additives, maintaining constant density during mixing, and avoiding delays during pumping. Additionally, avoid water-based fluids in workovers after CO_2_ injection begins, if possible.^60^

Table 3 presents a summary of the invaluable insights gained from the two projects, detailing their key findings and the implications for future initiatives. Each project contributed significantly to understanding, and these learnings are essential for shaping our strategic approach moving forward.

**Table 3:** Summary of Lessons from the Kingsnorth CCS and Peterhead CCS Projects

**project**	**apprenticeships**
Kingsnorth CCS	• abandoned wells need to be located, exposed, reopened, and a full CO_2_-resistant abandonment needs to be carried out using CO_2_-resistant cement
Peterhead CCS	• in order to ensure safe and efficient CO_2_ injection and storage, it is necessary to evaluate the wells integrity under prolonged CO_2_ exposure during the injection and storage phases
	• evaluating each well’s original design parameters and the quality of the abandonment plugs set in them
	• during the assessment for changing the use of Goldeneye wells from hydrocarbon production to CO_2_ injection, the focus was on materials, casing design, cement, and pressure management
	• the majority of production casing strings used in the Goldeneye Platform is safeguarded by 13 Chrome (13Cr) material tubing. Even under wet conditions, CO_2_ corrosion is not a threat to 13Cr steel
	• in carbon steel tubulars, CO_2_ corrosion can be prevented by managing the water content of the CO_2_ to avoid free water formation and prevent wet excursions. To ensure this, the water content in CO_2_ should be kept below 20 ppmW
	• run CBL to better understand the cement’s current integrity
	• during dry CO_2_ injection, carbonic acid is not formed, eliminating the potential for chemical reaction with Portland cement
	• it is recommended that the cement quality and placement be evaluated by means such as CBL (Cement Bond Logging) and USIT (Ultrasonic Imager Tool)
	• ensuring good cement placement by using a good spacer, proper lead and tail, and good centralization to avoid voids in the cement
	• it is recommended to use balanced cement plugs to avoid stringers and channeling for abandonment plugs
	• avoid using water-based fluids in workovers once CO_2_ injection has commenced, if possible

The REX-CO_2_ project (Reusing Existing Wells for CO_2_ Storage Operations) was a French initiative from September 2019 to August 2022 focused on carbon capture, utilization, and storage (CCUS). It aimed to evaluate the potential of repurposing oil and gas wells for CO_2_ storage and to develop a publicly available well-screening tool. This tool employs a qualitative screening method, assessing well design, integrity, and suitability for CO_2_ operations. It identifies the risk of CO_2_ leakage through the cement barrier and determines if a well is suitable for injection, monitoring, or production. Using a traffic light system, the tool indicates well conditions: green for good condition, yellow for moderate remediation needed, gray for missing vital information, orange for significant remediation required, and red for unsuitable wells. This user-friendly tool also provides actionable recommendations for well improvement.^62^

The REX-CO_2_ tool provides an overview of remediation actions needed for each well, including reusability assessments. This data aids decision-makers in cost-benefit analyses and project strategy. Savings from reusing existing infrastructure should be evaluated case by case, factoring in the costs of drilling new wells and remediation, which vary by well condition. The tool screens the current status of wells but is not a substitute for engineering assessments; it identifies wells suitable for further evaluation.^62^

The project, involving The Netherlands, the United States, Norway, the United Kingdom, France, Romania, and the United Arab Emirates, uses a well-screening tool to evaluate safe and economical CO_2_ storage. The framework will be validated to create customized reuse processes for different well designs at various sites. The tool has been applied to both onshore and offshore wells with depths of 1400–5000 m, covering diverse reservoir types, including gas and oil fields and saline aquifers.^62^

Wells were assessed in case studies across North America, Europe, and Asia, focusing on out-of-zone injection, structural integrity, barriers for well integrity, and material compatibility. The objective was to validate the developed tool.^62^

In addition, it is crucial to highlight the success of the Ravenna CCS project, which is currently in phase 1. It is Italy’s first carbon capture and storage initiative and the first in Southern Europe. Developed by Eni and Snam, it aims to support decarbonization in Italy and Europe by securely transporting and storing over 500 million tons of CO_2_ in depleted offshore gas reservoirs in the Adriatic Sea. Operations began in August 2024, utilizing existing infrastructures, which led to significant cost and time savings. Phase 1 has a maximum injection capacity of 25,000 tons of CO_2_ annually from Eni’s Casalborsetti gas treatment plant into the Porto Corsini Mare Ovest gas field. Phase 2, set to launch before 2030, will integrate multiple industrial emitters with a target storage capacity of up to 4 million tons of CO_2_ per year, potentially increasing to 16 million tons annually after 2030.^63^

## 6 Norm and Recommendation

### 6.1 ISO 27914/2017—Carbon Dioxide Capture, Transportation and Geological Storage—Geological Storage

There is one international standard specifically for CO_2_ storage well designs, ISO 27914. Its purpose is to provide safe and effective guidelines for storing CO_2_ in geologic formations throughout a project’s life cycle, applicable to both onshore and offshore sites. The standard is backed by various operational experiences from pilot to commercial-scale projects, focusing on well recommendations for CO_2_ storage.

These initiatives often employ methods from the oil and gas industry, especially CO_2_-enhanced oil recovery. Importantly, ISO 27914 is tailored for CO_2_ injection specifically for storage, not for hydrocarbon recovery or associated activities. It does not address postclosure guidelines or requirements and excludes disposal of acid gases other than those in the CO_2_ stream or underground storage methods like coal, basalt, shale, or salt caverns.

ISO 27914/2017 presents important information about well infrastructure and suggests evaluating elements that may affect the integrity of the materials and, consequently, the structure of the well. One critical element is the state and composition of the CO_2_ stream, which determines the selection of materials. Dry CO_2_ is usually not corrosive to carbon steel, but the presence of free water in the CO_2_ stream can create carbonic acid that corrodes carbon steel. Other constituents can also make a CO_2_ stream corrosive. Potential corrosive constituents of the CO_2_ stream, such as free water, NO_*x*_, SO_*x*_, H_2_S, and O_2_, must be identified to establish material requirements. Carbon steel can be used in CO_2_-handling equipment if the CO_2_ is free of water and corrosive components. Corrosion-resistant materials that require chemical treatment to maintain mechanical integrity are needed if corrosive components are present. Elastomer selection should be based on CO_2_ characteristics, and elastomers should be chemically and mechanically stable in the presence of CO_2_. Furthermore, corrosion rates increase significantly when CO_2_ is in a supercritical state (dense phase).

Determining the compatibility of injection stream components with the current storage unit environment is paramount to prevent any harm to the wellbore and its components. Laboratory modeling or geochemical simulation may be employed to guarantee compatibility. Chemical programs should also be designed to prevent internal and external corrosion of steel components. It is also advised that the CO_2_ stream composition be assessed regularly, preferably at least once a year, to gauge the quantity of CO_2_ injected and evaluate the injection system’s efficiency. When evaluating the physical properties of CO_2_, it is important to consider the range of pressure and temperature to determine the relationship between them in order to obtain the different CO_2_ phases, such as gas, subcritical gas/liquid, and supercritical liquid.^64^

According to Mahlobo et al.,^65^ CO_2_ behaves as a liquid with liquid-like properties when subjected to a pressure of 73.82 bar or higher and a temperature of 31.1 °C. Below 73.82 bar and 31.1 °C, CO_2_ is considered subcritical, possessing both gas and liquid properties. On the other hand, CO_2_ is in a gas phase when subjected to pressures between 1 to 50 bar and temperatures below 25 °C. Design temperatures should carefully consider where CO_2_ cooling might occur due to blowdown or large pressure drops causing Joule-Thompson effects from expansion.

When constructing a well, it is crucial to use cement that does not shrink during setting and can withstand temperature and pressure changes. The cement should also be resistant to chemical degradation from CO_2_. Adding elastomeric and fiber materials to the cement can improve the amount of deformation that cement can tolerate.^66^ The cement needs to create a hydraulic seal across the primary seal of the storage reservoir to ensure effective isolation. After placing the cement in the annulus, the cement sheath should be evaluated to detect any leaks. Pressure testing of the casing should be conducted only after the cement slurry has developed significant gel strength. The evaluation should confirm that the cement’s top aligns with the design depth. Other methods, such as wireline logs, can be used to determine whether the cemented annulus’s seal is adequate and has no leaks or defects. These logs detect flow behind the casing by measuring temperature, noise, and the flow of oxygen-activated water and CO_2_ molecules. Faulty cement sheaths will be repaired with suitable remedial methods and materials that meet the primary cementing design’s structural and sealing requirements.^64^

It is important to design all wells in a way that allows for continuous or periodic monitoring. This includes using logging and pressure testing equipment, such as wellhead, annulus, and downhole pressure and temperature gauges. Additionally, other equipment may be required to be run in the hole with tubing. For injection wells, a metering device should be associated with the well to monitor the mass of the fluid stream injected. It is also recommended that an injection temperature gauge be used to determine the density of the injected fluids, which allows for the estimation of bottom-hole injection pressures.^64^

Workovers and recompletions may be necessary to properly maintain or repair well components. If a well has significant mechanical defects such as cement cracks and fractures, cement debonding, surface casing vent flow, sustained casing pressure, gas migration, casing failure, and is likely to come into contact with injected CO_2_ or elevated pressure zones in the near to midoperational term, it must be remediated. However, remediation for other wells may be postponed until necessary conditions arise. Regarding legacy wells within the area of review, they shall be evaluated as potential leakage pathways. Therefore, it is important to identify wells that penetrate the storage unit, determine their status, characterize their construction type and mechanical defects, evaluate their potential to leak, and determine the chemical composition of well materials that will be exposed to a CO_2_-charged fluid. These are some of the key points to consider in the evaluation process.^64^

Before converting wells for CO_2_ storage, inspecting and testing the long-string casing for its integrity along its entire length is crucial. This can be done by obtaining and evaluating cement integrity logs, running and evaluating a casing inspection log for casing corrosion or damage, and pressure testing the casing in accordance with field pressure testing techniques without damaging the cement or cement-casing bond. Additionally, a baseline saturation log should be obtained to determine the gas saturation near the wellbore.^64^

After recompletion and workover activities, testing and evaluating the wellbore integrity is important to ensure that it meets the necessary requirements. Any casing leaks must be repaired, and if flow is detected along the casing, the cement integrity should be re-established. If fluid movement is detected outside the casing, the cement integrity should be established before a liner or casing patch is installed. In such cases, the options for repairing the casing may be limited to those not requiring pipe replacement, such as installing expandable liners and cement or chemical sealant squeezes. The casing should be pressure-tested after any casing repairs are made. The packer and tubing should be rerun into the well, and the mechanical integrity should be re-established by pressure testing the tubing/casing annulus. If the well cannot be repaired, it should be plugged and abandoned.^64^

All abandoned wells, including legacy wells, must be assessed using all available records to determine their history. This includes information on how the well was plugged and whether the method of plugging was effective in protecting and isolating potential CO_2_ storage units, preventing leakage, and ensuring that the surface is restored to its near-original condition. If the well cannot be identified, and there are no records available regarding how it was plugged, or the method of plugging did not meet the objectives, then the well must be carefully reviewed, assessed for leak risk, and qualified for the intended function of the storage project.^64^

## 7 Other Challenges

Geomechanical damage can significantly affect well integrity in CO_2_ storage fields. A thorough understanding of the geological and geomechanical characteristics of both the injection unit and the confining layer is crucial for evaluating the potential for CO_2_ leakage through natural features and induced fractures. The geomechanical processes related to CO_2_ injection can compromise the integrity of a CO_2_ storage site by creating or enlarging flow pathways due to fault reactivation, induced shear failure, and hydraulic fracturing. In all these scenarios, factors such as the in situ stress regime, changes in pressure and temperature, and the properties of the rock play a vital role in assessing the likelihood of CO_2_ leakage.^38,67^ While concerns regarding geomechanics were not the primary focus of this study, 26% of the references reviewed highlighted the importance of addressing this issue to reduce uncertainties surrounding well and field integrity in CO_2_ geological storage.

A significant challenge related to CO_2_ storage is the lack of knowledge about the current and future conditions of wells, particularly those that are suspended or abandoned. Evaluating these wells heavily depends on the availability and quality of data. Without comprehensive records detailing operational history, casing integrity, cement quality, and long-term material degradation, it becomes challenging to assess whether these wells can safely meet the new operational requirements for CO_2_ storage. Additionally, inconsistent historical documentation and potential structural deterioration raise concerns about leakage risks and the possibility of containment failure. While this issue is not the primary focus of the study, it is important to note that 19% of the references reviewed emphasized the necessity of addressing these challenges to reduce uncertainties surrounding well integrity in geological CO_2_ storage.

## 8 Results

Ensuring the long-term integrity and safety of CO_2_ injection and storage operations is paramount. Key factors such as cement integrity, corrosion risks, CO_2_ flow composition, and material requirements have gained significant relevance and are the focus of extensive research.

Based on the extensive literature review and lessons learned from field projects, we consolidated the most critical technical parameters for well screening into a comprehensive table (Table 4). This table serves as a practical guide for engineers and decision makers in the initial assessment of the feasibility of reuse, complementing the qualitative workflow presented in Figure 3. The parameters are categorized to facilitate systematic evaluation and replication of the methodology.

**Table 4:** Screening Parameters and Recommended Criteria for Evaluation of Wells for Reuse in CO_2_ Storage

**category**	**critical parameter**	**criteria for approval/recommended action**	**source in the manuscript/reference**
cement integrity	• cementation data	• cement integrity data (e.g., CBL, USIT) must be available and demonstrate hydraulic insulation. If unknown, should be acquired.	• Section 8.1, Figure 2,^58^
	• cement composition	• use of CO_2_-resistant cement (e.g., with elastomeric additives or fibers) is recommended, especially if there is a risk of water presence.	• Section 8.1,^35,65^
corrosion risk	• casing conditions	• inspection should reveal minimal or absent corrosion. Corroded casings should be replaced by corrosion-resistant materials (e.g., 13Cr steel, CRA).	• Section 8.2, Figure 2,^22,58^
	• history of exposure	• wells exposed to long periods of corrosive water flow should be inspected strictly.	• Section 3 (Power et al.,^12^)
CO_2_ stream composition	• water and contaminants (H_2_S, O_2_, SO_*x*_, NO_*x*_)	• should be kept below 20 ppm for dry injection, preventing the formation of carbonic acid. Corrosive constituents should be identified and mitigated to define material requirements.	• Section 6.1,^58,60,63^
material requirements	• material compatibility	• tubing, packers, and seals must be compatible with CO_2_.	• Table 1, Section 8.3,^11,22^
	• Galvanic corrosion	• should be prevented by using cathodic protection or avoiding dissimilar metals in contact.	• Section 8.3,^63^
context of the well	• well status	• active wells are preferable; abandoned wells require more complex and costly evaluation.	• Section 4,^1,6^
	• depth and location	• must intercept the storage reservoir and be located away from geological faults.	• refs 50,51
reservoir conditions	• pressure and temperature	• the casing design should be checked to withstand the new injection pressure and temperature conditions.	• Section 4,^58^
	• petrophysical data	• porosity (>20%), permeability (>100 mD), and reservoir thickness (>50 m) are positive criteria.	• ref 50

**Figure 3:**
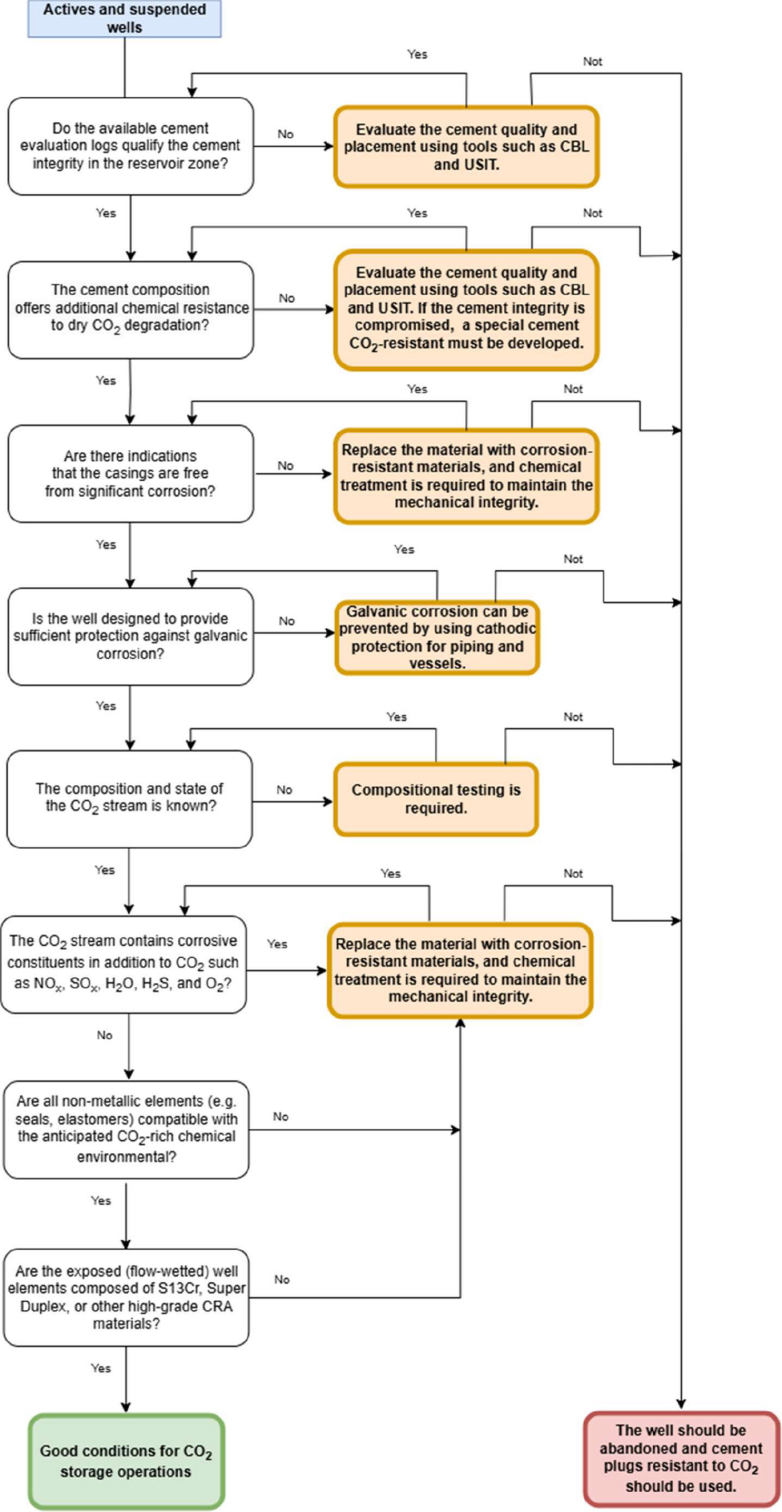
Workflow for evaluation of active and suspended wells, based on the critical parameters detailed in Table 4.

Figure 3 illustrates the workflow applied to both active and suspended wells. It is important to emphasize that this workflow is only relevant when the wells in question are nearing the reservoir designated for CO_2_ injection. If a well cannot be repaired using the recommended actions outlined in the orange boxes, it must be plugged and abandoned. For optimal safety, cement plugs resistant to CO_2_ are the recommended solution in such scenarios.

### 8.1 Cement Quality

The initial criteria are related to cement availability data and composition. First, having information about the current state of cementation is crucial to determine whether the well can continue to operate safely. If this information is unknown, it must be provided, or the well must be abandoned. In turn, the concern about cement compositions is allied to the potential risk of carbonation and it is strongly related to CO_2_ composition and consequently corrosion. The previous understanding that dry CO_2_ injection prevents chemical reactions with Portland cement has been challenged by recent evidence. As demonstrated by Zhu et al.,^68^ exposure to dry supercritical CO_2_ alone is sufficient to actively degrade cement, leading to a significant increase in permeability and a marked reduction in mechanical strength over time. This degradation occurs because the dry CO_2_ interacts with the cement’s microstructure, independent of bulk water. Furthermore, as noted by Fan et al.,^69^ drying the CO_2_ stream does not mitigate all corrosion pathways, as acidic impurities and other mechanisms remain active. Therefore, while dry conditions may slow certain reaction kinetics compared to a wet environment, they do not eliminate the fundamental risk of cement carbonation and property degradation. However, water may be present around the wellbores later in the wells’ life, leading to carbon acid degradation.

Organic polymers and nanoparticles can significantly enhance cement’s durability and CO_2_ corrosion resistance. These additives function by reducing permeability, improving bonding properties, and forming a protective barrier against corrosive agents. However, performance varies considerably across studies, with reported carbonation depth reductions ranging from 10 to 80%, making it difficult to conclusively identify a single optimal additive. Among organic polymers, WER and ANL appear to offer the most substantial reduction in corrosion depth.^70^

Notably, geopolymers present a highly promising alternative, demonstrating superior performance in several key areas compared to organic polymers. They exhibit greater resistance to acid attack, enhanced mechanical capabilities at high temperatures, and reduced shrinkage. For instance, one geopolymer sample showed a corrosion depth of only 0.3 mm after two months of exposure to a pH = 3 acid solution, whereas cement modified with organic polymers exhibited depths of 1–3 mm. Furthermore, low-calcium systems such as zeolites and other geopolymers have been found to possess greater resistance to CO_2_-rich brine than organic polymers. A primary operational challenge for geopolymers, however, is their short setting time, which can compromise pumpability in deep, high-temperature wellbores.^70^

Therefore, it is recommended to use a CO_2_-resistant cement. Randhol and Cerasi^66^ suggest that incorporating elastomeric and fiber materials into cement can enhance its ability to withstand deformation. Cement must form a hydraulic seal over the primary seal of a storage reservoir to ensure effective isolation. Some special cement has already been developed, but its composition is controlled and often kept confidential by the supplier.^40^

On the other hand, recent experimental studies consistently demonstrate the superiority of Calcium Aluminate Cement (CAC) over ordinary Portland cement (OPC) under CO_2_ storage conditions. Under simulated CCUS conditions (e.g., 150 °C and 10 MPa), CAC exhibits significantly lower compressive strength degradation (30.6%) compared to OPC (38.3%), alongside higher bearing capacity and a smaller crack area, indicating superior mechanical integrity under stress and corrosion.^71^ Microstructurally, CAC develops final porosity and permeability that are drastically lower than OPC after corrosion, a behavior attributed to the resistance of its hydration products to CO_2_ attack.^71^ Advanced CAC formulations modified with additives like hollow glass microspheres (CAC-μ) confirm this performance, exhibiting near-impermeable behavior, higher postexposure compressive strength, and a substantial reduction in carbonation compared to OPC-based systems.^72^ The transformation of phases assisted by additives contributes to pore-filling, reinforcing the microstructural density and solidifying CAC as a high-integrity sealing material for CO_2_ injection wells.^72^

In addition, polymer-based solutions like modified epoxy resin offer a robust defense against acid corrosion. A study investigating a water-based modified epoxy resin as an additive for oil well cement demonstrated a dramatic improvement in performance under simulated high-acid downhole conditions. After 30 days of exposure to aggressive liquid and gas-phase acidic media, the cement with epoxy resin exhibited remarkably low strength loss rates of 9.8 and 1.2%, respectively, and shallow corrosion depths of 3.1 and 10.6 mm. This performance was vastly superior to unmodified cement, which suffered strength losses of 32.1 and 17.6% with corrosion depths of 16.1 and 36.6 mm under the same conditions. The mechanism of protection was identified as a dual action: the resin fills the pore structure to improve compactness and forms a continuous, protective three-dimensional polymer film that effectively isolates the cement matrix from the corrosive acidic medium.^73^

### 8.2 Corrosion

Continuing with the process, the focus is on preventing corrosion in the casings. This issue is closely linked to both the quality of cementation and the composition of the fluid. If the casings display noticeable corrosion, it is necessary to replace the material with corrosion-resistant materials. Moreover, if there are any corrosive components present, chemical treatment will be required to maintain the mechanical integrity of the casings.

### 8.3 CO_2_ Composition and Material Requirements

The composition of the CO_2_ stream is an important factor when considering the use of carbon steel in CO_2_ handling equipment.

Dry CO_2_ is typically noncorrosive to carbon steel. However, the presence of free water in the CO_2_ stream can cause carbonic acid formation that corrodes carbon steel. Additionally, other constituents in the CO_2_ stream can make it corrosive. It is necessary to identify potentially corrosive constituents of the CO_2_ stream, such as free water, NO_*x*_, SO_*x*_, H_2_S, and O_2_, to establish material requirements. The selection of elastomers should be based on CO_2_ characteristics, and they should be chemically and mechanically stable in the presence of CO_2_. Determining the compatibility of injection stream components with the current storage unit environment is paramount to prevent any harm to the wellbore and its components. Regularly assessing the composition of the CO_2_ stream is highly recommended, preferably at least once a year. This will help determine the amount of CO_2_ injected and evaluate the efficiency of the injection system. To control corrosion, it is also suggested to include external coatings and periodic visual inspections of the interior portion of all vessels.^64^

The galvanic corrosion between dissimilar metals is also a concern. The ISO^64^ states that it can be prevented by using cathodic protection for piping and vessels. The last criterion takes into account the flow-wetted and gives a recommendation to use 13Cr steel. Negligible corrosion was observed in 13% Cr components during hydrocarbon production.^60^

## 9 Conclusions

Factors such as cement integrity, material compatibility, CO_2_ stream composition, and corrosion risks have become increasingly central to recent research on geological storage, highlighting their critical role in the safe reuse of existing wells. It is important to mention that dry wildcat wells, often poorly documented or abandoned without adequate casing, need a systematic assessment of these factors, as it is even more decisive, as it determines whether well repurposing is viable or if permanent abandonment is required. The flowchart developed in this study integrates these variables, providing a framework to transform active and nonproductive wells into CCS assets, provided they meet stringent integrity and monitoring standards. Thus, successful reuse depends not only on physical infrastructure but also on the ability to anticipate and mitigate risks through a multidisciplinary approach aligned with best practices.

Reusing existing wells can serve as a strategic catalyst to significantly reduce project lead time-a critical factor given that new drilling projects currently average six years from concept to commissioning. Such extended timelines risk delaying the achievement of the global 2030 targets. By prioritizing the repurposing of qualified wells, operators can bypass time-intensive phases like permitting and drilling, while adhering to rigorous assessments of well integrity and compatibility. This approach not only streamlines deployment but also aligns with sustainable practices, turning idle infrastructure into enablers of rapid CCS scale-up.

Although ISO 27941/2017 provides valuable recommendations, it is crucial to consult other relevant regulations for more accurate information and to improve the assessment of well integrity.

The developed flowchart offers a significant potential for the initial evaluation of the well, taking into account the most relevant issues involving the integrity of the well. However, to ensure a more robust assessment, additional technical conditions should be considered, including assessment points for abandoned wells and to redirect wells for monitoring CO_2_ storage operations.
